# Estimation of Earthquake Early Warning Parameters for Eastern Gulf of Corinth and Western Attica Region (Greece). First Results

**DOI:** 10.3390/s21155084

**Published:** 2021-07-27

**Authors:** Filippos Vallianatos, Andreas Karakonstantis, Nikolaos Sakelariou

**Affiliations:** 1 Institute of Physics of Earth’s Interior and Geohazards, and UNESCO Chair in Solid Earth Physics and Geohazards Risk Reduction, Hellenic Mediterranean University Research Center, 73100 Crete, Greece; akarakon@geol.uoa.gr (A.K.); nsakellariou4@gmail.com (N.S.); 2 Department of Geology and Geoenvironment, National and Kapodistrian University of Athens, 15784 Athens, Greece

**Keywords:** earthquake, early warning system, seismic risk mitigation, Gulf of Corinth

## Abstract

The main goal of an Earthquake Early Warning System (EEWS) is to alert before the arrival of damaging waves using the first seismic arrival as a proxy, thus becoming an important operational tool for real-time seismic risk management on a short timescale. EEWSs are based on the use of scaling relations between parameters measured on the initial portion of the seismic signal after the arrival of the first wave. To explore the plausibility of EEWSs around the Eastern Gulf of Corinth and Western Attica, amplitude and frequency-based parameters, such as peak displacement (*P_d_*), the integral of squared velocity ($I_{V\;}^{2})$ and the characteristic period (τ_c_), were analyzed. All parameters were estimated directly from the initial 3 s, 4 s, and 5 s signal windows (t_w_) after the P arrival. While further study is required on the behavior of the proxy quantities, we propose that the $I_{V\;}^{2}$ parameter and the peak amplitudes of the first seconds of the P waves present significant stability and introduce the possibility of a future on-site EEWS for areas affected by earthquakes located in the Eastern Gulf of Corinth and Western Attica. Parameters related to regional-based EEWS need to be further evaluated.

## 1. Introduction

The increasing urbanization worldwide has led to the establishment of large metropolitan areas near major active faults (whether on land or offshore), which pose a serious threat to the human population and infrastructures. Currently, an accurate earthquake forecast method appears to be far from operational. Consequently, attention has shifted to Earthquake Early Warning Systems (EEWS) for reducing earthquake risk [1,2,3,4,5,6], offering, in real-time, information for the incoming destructive seismic waves. EEWS leverage the nature of the different seismic waves; since “P waves carry information while S waves carry energy” [7], the fast, yet low amplitude P waves are analyzed to determine the earthquake damage potential, mostly caused by the slower, high-amplitude S-waves.

Over the last two decades, due to rapid technological advances in the fields of signal processing and data transmission, effective techniques have been developed to analyze seismological data in almost real-time [7,8,9,10]. In this direction, real-time seismology integrates fast and instantaneous data broadcast systems with automatic signal processing, providing reliable estimates of earthquake parameters (location and magnitude) in the first few seconds during its occurrence [10]. Modern EEWSs can offer a few to tens of seconds of warning time for impending ground motions, allowing for mitigation measures in the short term [11,12]—such an approach is in use in Japan [2], Taiwan [13,14], Mexico [11], and the United States of America [15]. The information produced by the aforementioned systems can be used by civil protection authorities to minimize the damages and losses (human and economic) [16,17].

In most EEWSs, the peak amplitudes of displacement (*P_d_*), along with the integral of squared velocity ($I_{V\;}^{2}$) parameter estimated from the initial Pwave, scales with the earthquake magnitude and peak ground acceleration (PGA) [17,18,19]. This suggests that we can predict the earthquake magnitude and the intensity of the ground motion using the initial P waves. It is still under scientific debate whether determining the attenuation relationship of *P_d_* naturally leads to practical applications for early warning. We note that recently the time evolution of *P_d_* was investigated, suggesting a universal pattern of initial growth that is inconsistent with deterministic models of earthquake rupture [19]. Therefore, when a large earthquake occurs, its location can be quickly obtained from a few P wave arrival times at nearby stations, and *P_d_* can then be used to determine its magnitude via the attenuation relationship, which can then be used in the EEWS for emergency operations.

Earthquake early warning systems can mainly be divided into two categories: (a) on-site and (b) regional. The on-site EEWS use one seismic station, while the regional approach requires a seismic network [20]. This poses fundamental differences in the design, operation, and maintenance of the EEWS. In (b), the time between the event origin and the alert emission is reduced, as it requires P wave triggers in multiple stations. By contrast, in (a), a single arrival is adequate. The above has obvious implications on the robustness of the emitted alert and whether it is false or not. On the other hand, on-site approaches avoid pitfalls in the earthquake and magnitude estimation (which, in turn, do not affect the quality of the alert). The development and implementation of regional EEWSs were summarized in a number of previous studies [6,20], along with some very recent approaches that predict seismic intensity at a target site based on the seismic intensities observed at seismic stations around the target site [21]. This type of EEWS requires the arduous task of designing and deploying a network of sensors with specific geometry and specifications (as opposed to on-site systems that require either a single or a few instruments). In general, the discussion about EEWSs’ limitations is ongoing, with questions, such as the physical boundaries of warning times in relation to the level of ground motion, exist and are still open [22,23].

In this work, we analyze parameters that are used to determine earthquake magnitude in real-time for the areas of the Eastern Gulf of Corinth (EGoC) and Western Attica (WA). We focused on the correlation of (a) *P_d_* (as both a regional and an on-site proxy) with the magnitude M_w_ and the hypocentral distance H, (b) the characteristic period τ_c_ with magnitude, averaged over the area, (c) the integral of squared velocity ($I_{V\;}^{2}$) with PGA on-site and inter-site. The latter aims to investigate whether it is possible to use P waves near the source as a proxy for S-wave shaking at a far target. In that context, we also design relations between *P_d,v,a_* and PGA, for inter-site use. Evaluating the practicability of an EEWS in this area is justified by its high level of seismic hazard. The area spans two zones of the New Greek Seismic Code (NEAK) with expected PGA values at a minimum of~157 cm/s^2^ and a maximum of~353 cm/s^2^ for a return period of 475 years (see Figure 1 in [24]). It is noted that the NEAK features four broadly defined zones with wide PGA ranges. Ref. [24] using a more refined model and various empirical Ground Motion Prediction Equations found expected PGA between 100 cm/s^2^ and 300 cm/s^2^. The European Seismic Hazard Map (ESHM13) by [25] confirms this by categorizing the area as high-hazard with an expected PGA of ~300 cm/s^2^. Moreover, large oil refineries and power plants are located in the EGoC and the coast of WA. For them, suitable safety measures, such as automatic blocking of pipelines or gas lines to prevent fire hazards or the automatic shutdown of manufacturing operations to avoid valuable equipment exposure, could be adopted for damage reduction. Therefore, the introduction of an EEWS is a worthwhile objective because it can be effectively used to reduce the damage caused by the strongest earthquakes. It is noted that the effectiveness of an EEWS is multifaceted, with issues such as civil protection response [26] and network limitations factored in. In a recent feasibility study by [27], it was shown that events in the Eastern Gulf of Corinth could have a warning time of 10 s in Athens. We note that the Virtual Seismologist (VS) EEW software and PRESTo have been installed in the area of the Western Gulf of Corinth and Central Greece, and their initial performance has been presented [28,29].

## 2. Seismotectonic Setting, Data Acquisition, and Processing

The Gulf of Corinth (GoC) is an important E–W oriented neotectonic half-graben, bounded by normal faults, gradually evolving during the Pleistocene, as it is evidenced by the intense seismic activity [30]. Normal faults continue from the Eastern end of the GoC (EGoC) towards the Saronic Gulf [31].

**Figure 1:**
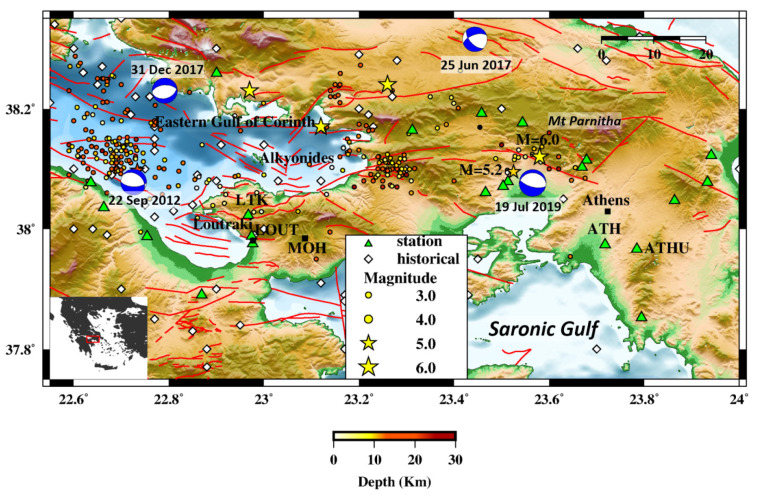
Geotectonic map of the broader area of the Eastern Corinth Gulf and Western Attica. Earthquakes marked by circles (for M_w_ < 5.0) and stars (M_w_ ≥ 5.0) (colored according to the included depth color bar) belong to the catalog used in the current study, obtained by the International Seisomological Centre (ISC), between 2008 and February 2021. Yellow stars indicate events between 1900 and 2008 along with historical earthquake locations (white diamonds) and faults (red lines) after [31] are presented. Focal mechanism solutions were acquired from the Seismological Laboratory of the National and Kapodistrian University of Athens (NKUA-SL). Sensor locations (triangles) and sites of high interest (squares) are also shown. Inset: Greece with the study area marked (rectangle).

There are two main sources of strong earthquakes in the area: (a) the Parnitha range in WA and (b) the Alkyonides Islands in the EGoC. Parnitha acts as a NW boundary to the Athens basin. Its, generally, E–W and NE–SW trending faults are extended southward and have been the cause of earthquakes that have had a detrimental impact to Athens and the surrounding area. On 7 September 1999, a M_w_ = 6.0 event occurred at the southern part of Parnitha, about 20 km NW of Athens. The ferocity of the shaking led to billions of dollars in damage, decimated infrastructure, as well as hundreds of casualties [32]. Its shallow focus and proximity to the capital [33,34], as well as issues with constructions on unsuitable sites [35], were the culprits for the high intensities observed. More recently, in 2019, a M_w_ = 5.2 earthquake occurred in the same structure [36], acting as a reminder of the danger posed by active faults in the vicinity of Athens (Figure 1); other than a partial collapse of an abandoned building, the city was left unscathed, even though its western outskirts sustained some damage [37]. The region NW of Parnithta also hosts faults that cause shallow seismicity (with potential moderate to strong events) and, consequently, could have an impact on Athens [38]. Another source lies closer, NE of Athens, but has, so far, witnessed some felt events, such as the M_w_ = 4.2 earthquake of 2018 [39], without any noticeable impact on hazard, due to the weak magnitudes.

In Figure 1, a geotectonic map of the broader area of the Eastern Corinth Gulf and Western Attica is presented where all the events between 1900 and 2008 [40] along with the historical earthquake locations [41] and the faults are presented.

The islands of Alkyonides were the sole focus of seismic hazards associated with Athens and the Eastern Gulf of Corinth until 1999. A series of strong events between February and March of 1981, with magnitudes of M_w_ > 6.0 for three of them, caused unprecedented damages in the area [42]. Further study of the 1981 area implied the occurrence of several other similar events (in terms of deformation) in historical times, indicating the region as a prime contributor to regional seismic hazard [41,43]. Since then, the absence of strong events or significant seismicity led to a sparse seismological network, despite the region’s importance. Either Parnitha or Alkyonides could produce an earthquake that affects an area that includes a major city (Athens), a significant tourist hub (Loutraki), and industrial infrastructure, such as the oil refineries of the Motor Oil Hellas (MOH) group (Figure 1).

To estimate the scaling parameters for an EEWS in the area, we retrieved an event catalog for the Eastern Gulf of Corinth and Western Attica from the International Seismological Center (ISC), consisting of 278 earthquakes between January 2008 and February 2021, with a minimum magnitude of 3.0 and a maximum depth of 30.0 km. The catalog was obtained through ISC’s European Integrated Data Archive (EIDA) node, using the respective international Federation of Digital Seismograph Networks (FDSN) service. To homogenize the magnitudes, we used the relations proposed by [43,44] for converting to M_w_. Then, we searched for waveform data in the archive of the Geodynamic Institute of the National Observatory of Athens (GI-NOA) through their EIDA node.Their database includes recordings provided by several Greek institutes, recorded by stations of the Hellenic Unified Seismological Network (HUSN) [45], and initially selected 33 suitable stations, including 24 velocimeters and 9 accelerometers. The GI-NOA node also includes detailed station metadata, such as the response functions of both velocimeters and accelerometers [46]. We set a maximum event search radius of 100.0 km around each station of interest. This set included two sites in Loutraki (LOUT and LTK) and two in Athens (ATH and ATHU). These sites feature long-term installations (ensuring a wealth of available data) and are located close to the two most important seismic sources around the local industrial infrastructure (e.g., oil refineries) and the metropolitan city of Athens, i.e., Alkyonides [41,42] and Parnitha [32,33,34,35,36,37]. All four stations have broadband sensors.

**Figure 2:**
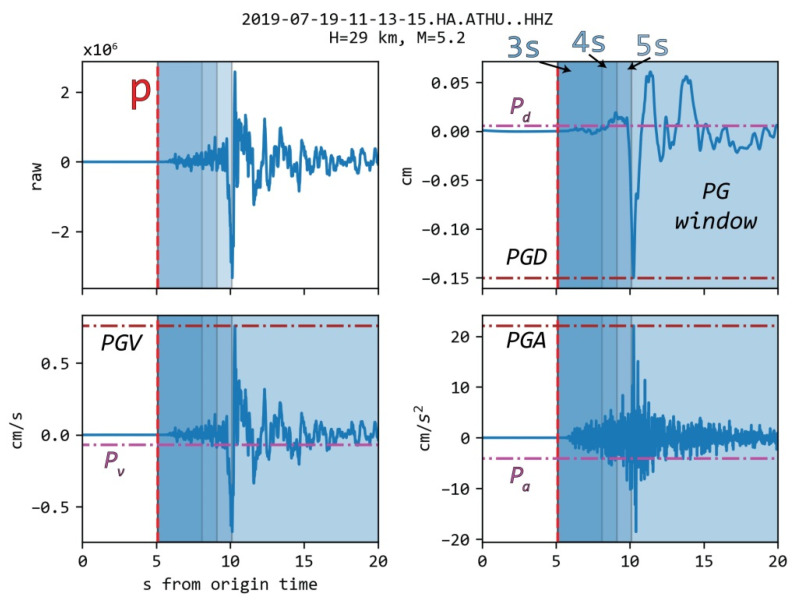
Vertical recording of the M_w_ = 5.2 July 2019 event at station ATHU (located in Athens, approximately 29 km from the epicenter). The raw (**upper left**), displacement (**upper right**), velocity (**bottom left**), and acceleration (**bottom right**) recordings are shown. The horizontal magenta line indicates the *P_d,υ,a_* measurement for a signal window of 3 s length, while the horizontal brown line showcases the determined peak ground motion (e.g., PGA for acceleration). The automatic pick is denoted by the vertical red dashed line and the S arrival by the vertical blue dashed line. The following signal windows are highlighted by the gradually less shaded areas (from darkest to brightest): 3 s, 4 s, 5 s, and peak ground measurements window. The hypocentral distance (H) and magnitude (M) are also noted.

Data acquisition and signal processing (including instrument response removal and transformations between different ground motion outputs) was carried out using routines by ObsPy (see https://docs.obspy.org, accessed on 26 July 2021) [47]. To process the data, we first ensured that no clipped recordings were included by visually inspecting suspicious event-station pairs (i.e., seismographs located within 30.0 km of earthquakes with a magnitude of at least 4.5). Then, we automatically picked the arrival of the P-phase with the algorithm proposed by [48]. To estimate any EEWS parameter, we used three time-windows starting from the P arrival and extending to 3 s, 4 s, or 5 s (t_w_). Since, in most cases, the used t_w_ is significantly larger than the expected automatic phase picking uncertainty, the results are expected to be largely unaffected by it. To remove noise that could lead to issues with the picker, we first applied a bandpass filter between 1.0 and 20.0 Hz. This filter was used exclusively in the picking process. Moreover, we estimated the S-P time with TauP [49] using a local velocity model [50]. The raw, unfiltered data were then deconvoluted with the instrument’s response to obtain the actual ground motions (i.e., displacement, velocity, and acceleration) at each site. After applying a high pass filter with a corner frequency of 0.075 Hz [51], we determined the peak value for each motion type (hereafter noted as *P_d,u,a_* for displacement, velocity, and acceleration, respectively) in the three t_w_, as well as the peak ground acceleration of the shear-waves (hereafter, called *PGA*) in a window ending 20 s after the theoretically estimated S arrival. However, if the signal window t_w_ exceeded the estimated S-P time, the measurement was rejected. We set the S-P time exceedance criterion to a priori avoid cases of overlapping windows for measurements of P peak and S peak amplitudes, as in the case of the t_w_ = 5 s window in Figure 2. The process was repeated for all available recordings and all three channels (Figure 2).

In the final dataset, we removed observations with a PGA to *P_a_* ratio less than 2.0. This criterion was important to avoid cases of erroneous picking, inherent instrumentation issues (e.g., clock drifts), and problems with the instrument correction (e.g., errors or omissions in the provided instrument response for each epoch). The final dataset contains 2308 measurements among the three time-windows, for 240 events and 23 stations (947 event-station pairs).

**Figure 3:**
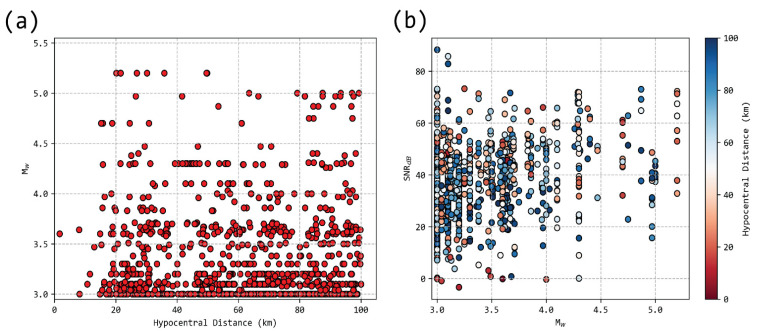
Distribution of available event-station pairs, shown by the relation of magnitude (M_w_) to the hypocentral distance (**a**) and SNR (in dB) as a relation of magnitude and the hypocentral distance (**b**).

Our observations span a magnitude range between 3.0 and 5.2 (Figure 3). There is a good distribution for lesser magnitudes; however, magnitudes above 4.5 present sporadic measurements, with large gaps, as in the case of the sole M_w_ = 5.2 event. Acceptable observations span a distance between ~20 km and ~50 km (Figure 3a). The signal-to-noise ratio (SNR, in dB), indicated an increase with magnitude (Figure 3b). However, we noted that the considered earthquakes were recorded in a variety of stations with different conditions. For example, two of the closest seismological stations to the M_w_ = 5.2 event were located in the city of Athens, largely affected by urban noise, which, as logic dictates, is a detriment to SNR.

## 3. Empirical Correlation Laws for Eastern Gulf of Corinth and Western Attica Region

Determinations of magnitude and the ground shaking intensity from the initial P wave are two important elements for earthquake early warning. The strength of shaking can practically be represented by PGA. Here we present the first results from an effort to establish the correlation laws required for regional EEWS (in Section 3.1 and Section 3.2) and that for on-site (in Section 3.3). We also explore the possibility of inter-site relations (in Section 3.4).

### 3.1. Peak Ground Displacement, Magnitude, and Distance

**Figure 4:**
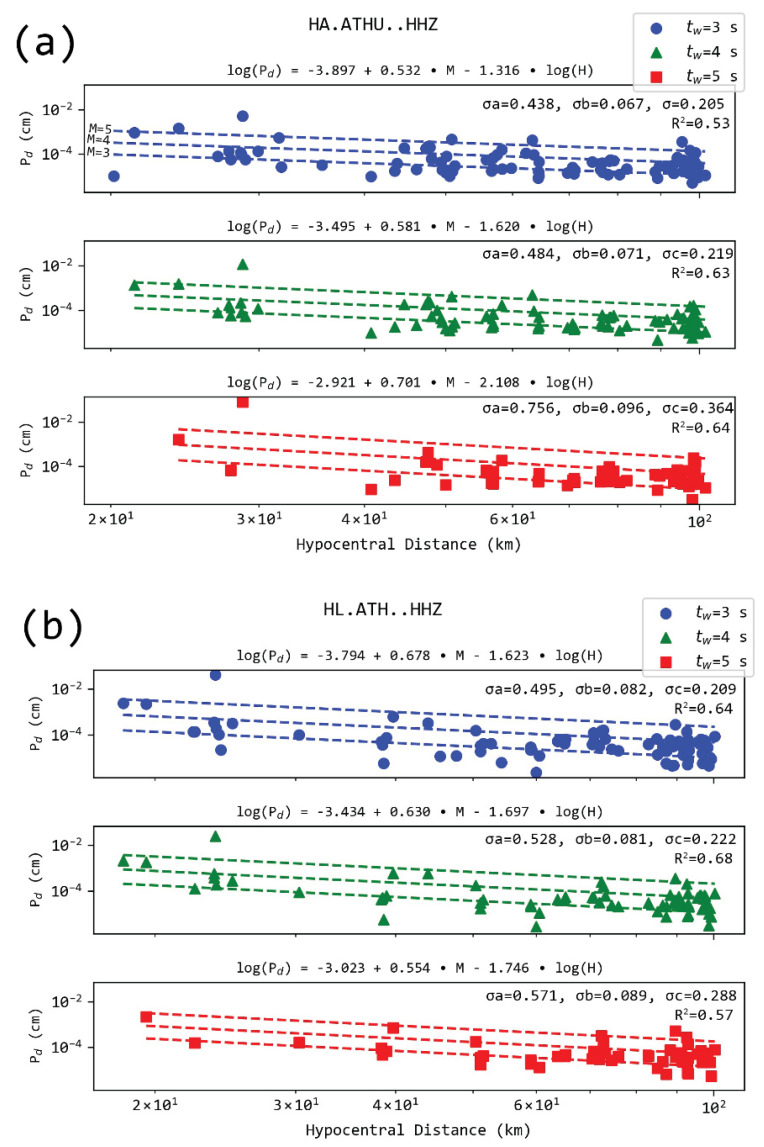
Regression analysis for *P_d_* measured in two stations located in Athens stations. (**a**) for ATHU and (**b**) for ATH and for 3 s (top), 4 s (middle), and 5 s (bottom) from the P arrival. The dashed lines show the model for M = 3.0, M = 4.0, and M = 5.0. σa: s.d. for the intercept, σb: standard deviation for the magnitude variable’s constant (M), σc: s.d. for the hypocentral distance (H) variable’s factor, and R^2^ the squared correlation coefficient for the regression.

In [2,52], based on the *P_d_* measured on the 3 s window starting from the *P* wave arrival, estimated the magnitude of a seismic event. Furthermore in [53,54] showed that *P_d_* scales with the earthquake magnitude and could be used for its real-time estimation in EEWS applications. Using the *P_d_* values estimated (in cm), we assume a simple linear regression model among the logarithm of *P_d_*, the magnitude *M* and the logarithm of the hypocentral distance *H* (in km) as:

(1) $$\log\left(P_{d}\right)=a+b*M+c*\log\left(H\right)$$

where *a*, *b*, and *c* are constants to be determined from the linear least-squares regression analysis. The resulting best fitting relationship for the two stations in Athens is presented in Figure 4 for the three signal windows.

For stations located in Athens (i.e., ATH and ATHU) and for the different t_w_ used, the mean relationships are:

(2) $$\begin{array}{c} \log\left(P_{d}\right)=-3.846+0.605*M-1.474*\log\left(H\right),{\;\mathrm{for}\;t}_{w}=3s\;\\ \log\left(P_{d}\right)=-3.465+0.606*M-1.659*\log\left(H\right),{\;\mathrm{for}\;t}_{w}=4s\\ \log\left(P_{d}\right)=-2.972+0.627*M-1.927*\log\left(H\right),{\;\mathrm{for}\;t}_{w}=5s \end{array}$$

Standard deviations of intercepts for *P_d_* relations in Athens ranged between 0.438 and 0.701. The magnitude’s factor deviation was between 0.067 and 0.096, while hypocentral-related constants were bounded by deviations within 0.205 and 0.364 (Figure 4). It is apparent that wider P wave windows yielded larger deviations. However, this cannot be considered as the sole metric of regression quality; thus, we also included the relevant correlation coefficients (Figure 4).

The regression constants are very close to the ones presented in [49] for Southern California. Regression results for the rest of the combinations between station and time windows are provided in the Supplementary Materials. We include 42 station-time window pairs with the corresponding regressions. The individual correlation coefficient and the number of observations used in the regression are also included.

**Figure 5:**
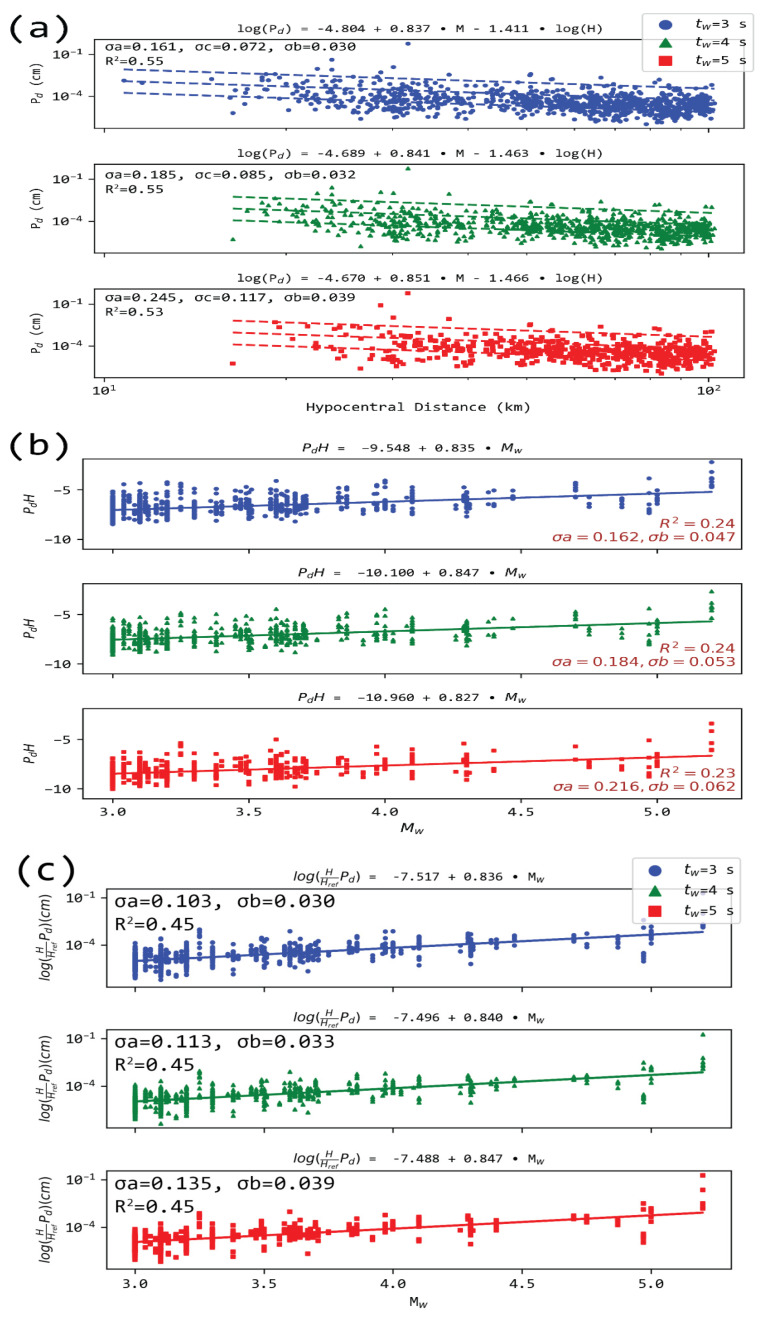
Regression analysis for (**a**) *P_d_* for the overall dataset and (**b**) $P_{d}H=\log\left(P_{d}\right)-c*\log\left(H\right)$, using c for each t_w_ as seen in panel (**a**,**c**) for the corrected *P_d_* for the overall dataset by a factor determined by the hypocentral distance and a reference distance *H_ref_* = 100 km. The σb refers to the coefficient of the magnitude variable. The rest of the notation as in Figure 3.

Performing a regression over the whole dataset yields the relations shown in Figure 5. Lower magnitudes seem to correspond to more scattered values of *P_d_*. Correcting the displacement by a factor determined by the hypocentral distance, as in [55,56], the relation $\log\left(P_{d}\right)-c*\log\left(H\right)=a+b*M$, exarcebates this issue (Figure 4b). Regressing for the two independent values (i.e., magnitude and distance) yielded a relatively higher correlation than the normalized ground amplitude approach (see Figure 4).

An alternative approach to correct the displacement by a factor determined by the hypocentral distance and a reference distance (in our case, *H_ref_* = 100 km) is presented in [55], where $\log\left(P_{d}\frac{H}{H_{ref}}\right)=\alpha Μ-b$, with α ≈ 0.84 and b ≈ 7.5, close to that presented in [56] for a regional EEWS application in Israel (Figure 5c), yielding a relatively higher correlation than the normalized ground amplitude approach (see Figure 4 and Figure 5).

**Figure 6:**
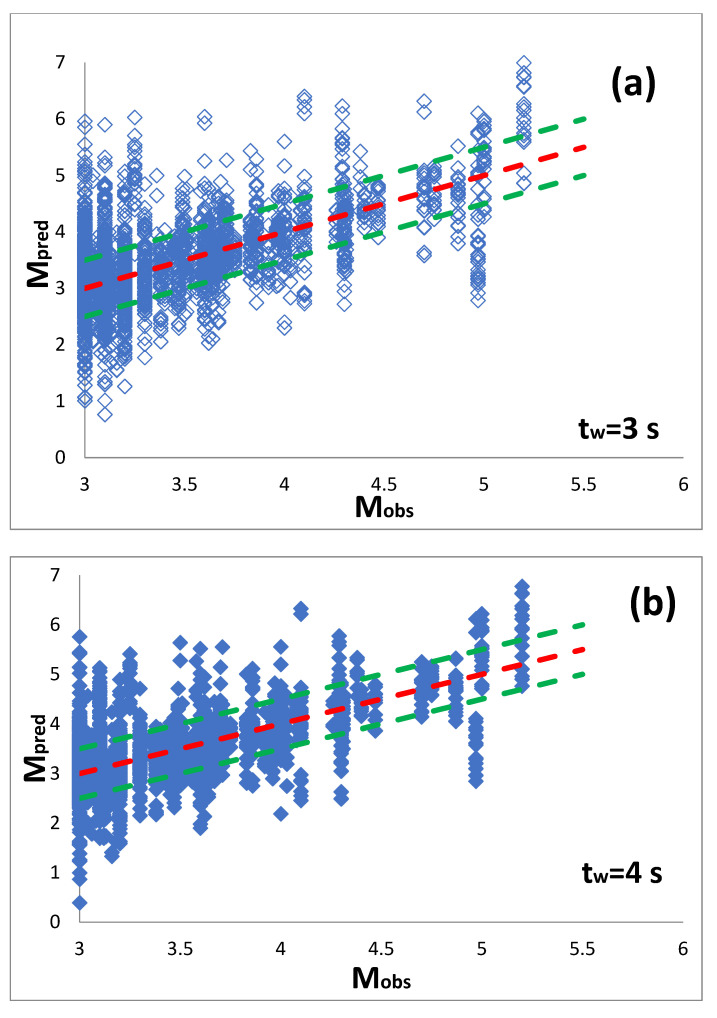

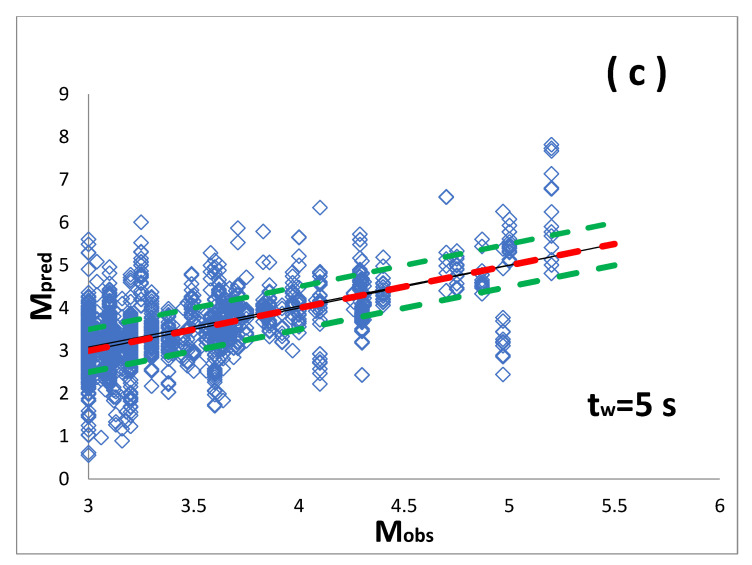
The predicted magnitude M_pred_ along with that observed M_obs_ for 3 s (**a**), 4 s (**b**), and 5 s (**c**) from the P arrival. The red line indicated the dichotomous while the two green lines are ±0.5 units from the dichotomous, respectively.

To evaluate the predicted magnitude M_pred_ using Equation (2), we plotted it along with that observed M_obs_ (Figure 6). In this plot, the dichotomous line is presented. We observe that for all used three time-windows starting from the P arrival (i.e., t_w_ 3 s, 4 s, and 5 s), the majority of M_pred_ values were within ±0.5 units from that observed. Furthermore, the distribution of the ratio M_pred_/M_obs_ and the deviation M_pred_-M_obs_ are presented in the Appendix A. Table 1 summarizes the average values indicate that the ration M_pred_/M_obs_ is close to one while the deviation M_pred_-M_obs_ close to zero for the t_w_ used.

### 3.2. The Characteristic Period τ_c_

In Ref. [7] developed a different parameter called characteristic period (τ_c_), which is an expression of the ratio between velocity and displacement records. This parameter is determined as follows: first, we compute $r=\frac{\int_{t_{p}}^{t_{p}+t_{w}}{\dot{u}}^{2}\left(t\right)dt}{\int_{t_{p}}^{t_{p}+t_{w}}u^{2}\left(t\right)dt}$ where *u*(*t*) is the ground motion displacement and $\dot{u}\left(t\right)$ the velocity. The integration is taken in the time window t_w_ after the arrival time t_p_ of P waves, then $\tau_{c}=\frac{2\pi }{\sqrt{r}}$ in s. Data from both velocity and acceleration recorders have been used to estimate τ_c_ in several seismic regions, demonstrating that the characteristic period scales with seismic event magnitude [6,57,58,59,60]. Up to a few hundred kilometers from the seismic source, it is independent from the epicentral distance [6]. We note that theoretical models document the characteristic period scaling with source properties under the assumption of a constant stress drop [61].

**Figure 7:**
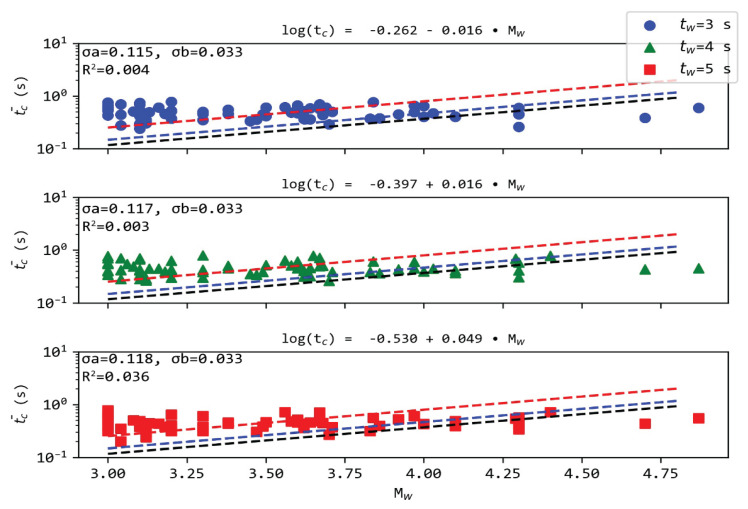
The characteristic period τ_c_ estimated for different magnitudes M. The dashed lines are the theoretical expressions for stress drop 1 (red), 5 (blue) and 10 (black) MPa, respectively. The rest of the notation as in Figure 3.

We plotted the τ_c_, averaged for all available measurements per event in hypocentral distances within 60 km and 100 km (Figure 7). We rejected average t_c_ values obtained from less than four observations (e.g., [51]). This led to the exclusion of some events, such as the M_w_ = 5.2 earthquake of our dataset. Our results suggest an absence of correlation between τ_c_ and the earthquake magnitude M_w_ (Figure 7). A possible reason could be the variance of stress drop in the earthquakes used in the data set as indicated in Figure 7, where the theoretical expressions for stress drop at 1, 5, and 10 MPa have been added [58]. One more reason for the τ_c_ scatter could be the SNR. In case of very weak displacement (e.g., in weaker earthquakes), the increased noise content can have an adverse effect on τ_c,_ causing the observed scattering. However, the use of broadband velocity recorders can reduce the SNR of strong motions, as weak signals of a wider spectrum contribute to the final waveform (as opposed to recordings of accelerographs, which commonly feature a narrower flat response).

### 3.3. By-Passing the Earthquake Magnitude Estimation: The Integral of the Squared Velocity ($I_{V}^{2}$)

In Ref. [62] first introduced the use of the integral of the squared velocity ($I_{V\;}^{2}$) estimated from *P* wave time windows in EEWSs for real-time magnitude estimation. $I_{V\;}^{2}$ is defined as: $I_{V\;}^{2}=\int_{t_{p}}^{t_{p}+t_{w}}\upsilon^{2}\left(t\right)dt$. The presented parameter is in cm^2^/s. $I_{V}^{2}$ has been adequately correlated with earthquake magnitude [62], as well as Peak Ground Velocity (PGV) and macroseismic intensity [17]. We identified a stable correlation between $I_{V\;}^{2}$ and PGA as reported at the same station [17]. Consequently, the quantity could be used as a proxy for rapid damage assessment. The relationship between $I_{V\;}^{2}$ and PGA could therefore be used to identify in real-time and before the arrival of S waves whether a site is going to be adversely affected or not, and, thus, has the potential to become key in the design of on-site EEWSs, assisting civil protection in acting immediately, according to the severity of the situation.

**Figure 8:**
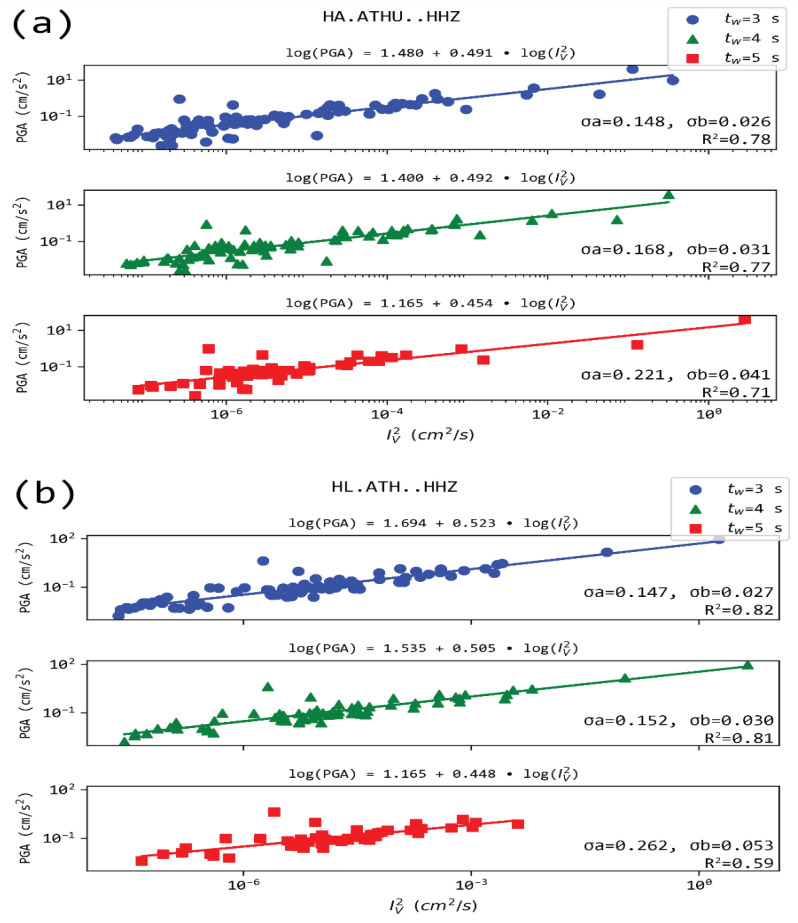
Regression models of PGA and $I_{V\;}^{2}$ for the two stations located in Athens, i.e., ATHU (**a**) and ATH (**b**), for the three time-windows (t_w_) after the P arrival. The rest of the notation as in Figure 3.

**Figure 9:**
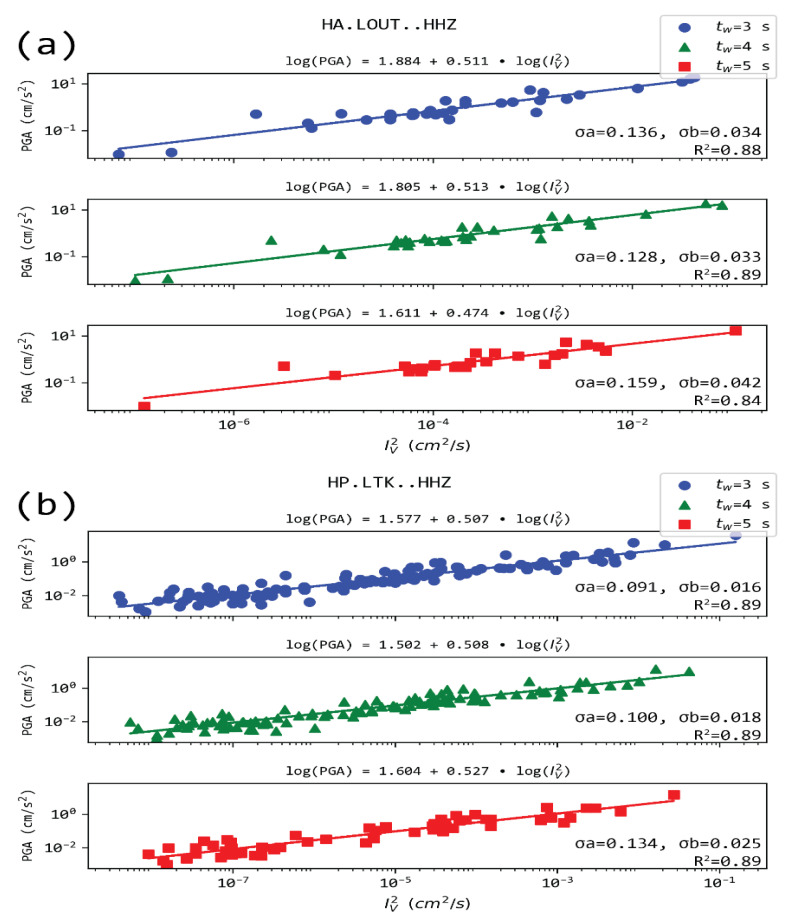
Regression models of PGA and $I_{\upsilon \;}^{2}$ for the two stations located in Loutraki, i.e., LOUT (**a**) and LTK (**b**), for the three time-windows (t_w_) after the P arrival. The rest of the notation as in Figure 3. The logarithms of the two quantities show a very high correlation.

Figure 8 and Figure 9 present PGA as a function of $I_{V\;}^{2}$ in the case of stations LOUT and LTK, located in Loutraki, close to the epicenters of the Alkyonides (1981) earthquakes, and ATH and ATHU stations located in Athens, close to the Parnitha fault, which recently activated (2019). For all the cases, a correlation of the form: $\log PGA=a+b*\log\left(I_{V}^{2}\right)$ exists for all time windows t_w_ used, with values in the range 1.8–2.5, while b is quite stableat ~0.5 (the values range between 0.48 to 0.52). It is noted that while $I_{V\;}^{2}$ is computed from measurements in the vertical channel, PGA refers to the maximum observed acceleration between the two horizontal components of the sensor. Regression coefficients for this relation in 69 station-time window pairs are provided in the Supplementary Materials, along with the respective correlation coefficients and number of observations used in the analysis.

### 3.4. PGA in Target Site versus P_d,v,a_ and $I_{V}^{2}$ Estimated Close to Epicenter

**Figure 10:**
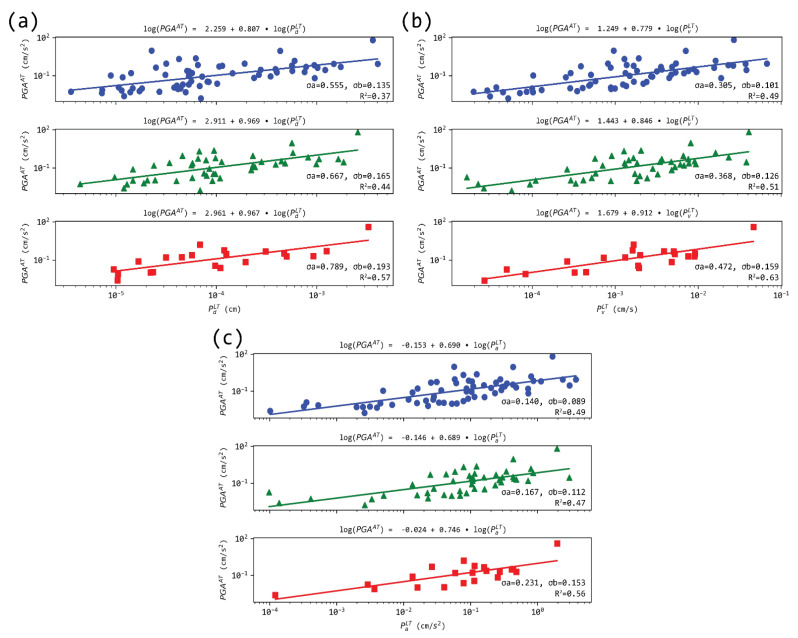
Regression models between PGA measured in Athens (AT) and peak displacement *P_d_* (**a**), velocity *P_v_* (**b**), and acceleration *P_a_* (**c**) measured in Loutraki (LT), for the three t_w_. Notable scatter is observed, compared to the individual station analysis. The rest of the notation as in Figure 3.

While previous works [54,57] have discussed the integration of an on-site approach to a larger regional-based model, by using a first-trigger site near the source to issue an area-wide alert, we investigated the possibility of establishing scaling laws between specific sites. If this proves successful, inter-station relations could be used to estimate the shaking (e.g., PGA) at a target site from a proxy recorded at a near-source receiver (e.g., *P_d_* or $\;I_{V}^{2}$). This would remove some of the obstacles raised by regional EEWSs, while, at the same time, removing the main drawback of on-site methods (i.e., being able to predict the ground motion at the site that also evaluates the proxy parameter). In Figure 10, the PGA values, recorded in the stations of Athens, are plotted as a function of *P_d_*, *P_v_*, and *P_a_* measured from records three t_w_ as estimated in the stations of Loutraki located in the vicinity of Alkyonides (1981) strong events. Figure 10 shows that, overall, the PGA values increase logarithmically with *P_d_* in the investigated range of magnitudes. Moreover, P_*v*_ and P_*a*_ follow a similar pattern. However, correlations are not satisfactory; at all combinations, but one, R^2^ stands below 0.60.

**Figure 11:**
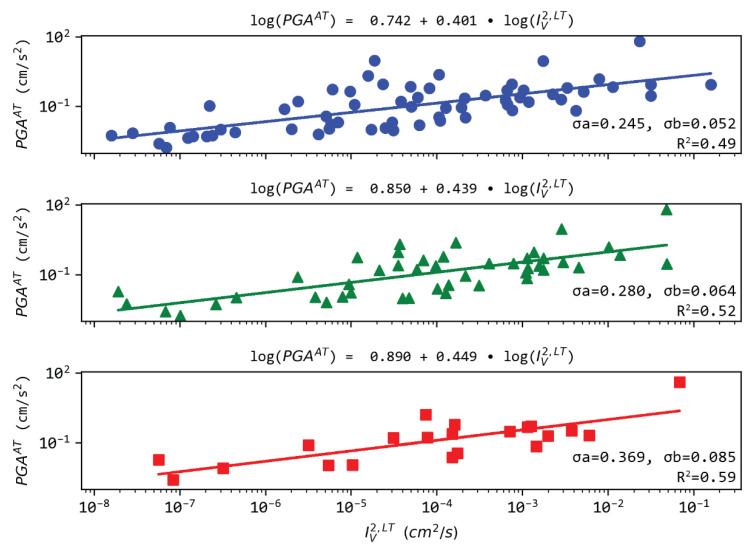
Regression analysis between PGA measured in Athens (AT) and $I_{V\;}^{2}$ in Loutraki (LT). The rest of the notation as in Figure 3.

Similarly, we explored the relation between PGA in Athens and $I_{V\;}^{2}$ in Loutraki (Figure 11). A clear logarithmic dependence exists with a slope in the range 0.40–0.45. We note that slopes are in agreement with the on-site cases in Section 3.3. Correlation is similar to relations shown in Figure 10, ranging around 0.50.

## 4. Conclusions

Today, the development of EEWSs represents one of the most useful strategies to mitigate seismic risk in short timescales, and several countries worldwide are promoting and developing such systems [2,11,13,14,15,16,60]. In the context of seismic risk management, they are considered a reasonably cost-effective solution for loss reduction.

In this study, we estimated empirical scaling relationships between the EEW parameters *P_d_* with magnitude and hypocentral distance and τ_c_ with magnitude, using measurements from stations all over the region of EGoC and WA. Moreover, we established on-site relations between $I_{V}^{2}$ and PGA. Finally, we explored the laws between stations located near the EGoC sources and stations in Athens to estimate inter-site relations for *P_d,v,a_* or $I_{V}^{2}$ (recorded at the former) and PGA (recorded at the latter).The data have been acquired by stations of the active seismological network in the area (HUSN),whose distribution ensured a good distance and azimuthal coverage.

Using an amplitude-based parameter (*P_d_*), an appropriate attenuation relationship was derived (see Supplementary Materials, where the goodness-of-fit parameters are given, too). The relationship of *P_d_* with the magnitude seems to be less scattered than that of τ_c_ (Figure 5 and Figure 6). However, to compute the magnitude from *P_d_*, a quick and reliable estimate of the distance is also required. In other words, the suitability of *P_d_* as an EEWS parameter is directly related to the quality of the rapidly estimated earthquake location. The estimated *P_d_* versus M and H relationship is of the same form as those obtained from data of various regions where EEWS operate but with different scaling coefficients, possibly suggesting that, despite the scatter of the data around the mean, the correlation of *P_d_* with M and H depends on the geological conditions [59,63,64]. This linear relationship persists throughout the entire magnitude range in our study. We note that these relations were constructed for a small magnitude range and further work is needed to adapt it to large earthquakes.

A statistically significant relation between log(τ_c_) and the magnitude could not be identified, since a low correlation appears (Figure 6). This could be attributed to small P amplitudes or high noise content in broadband stations or the variance in stress drop by the earthquakes of different sources [61].

Our results uncovered high correlation between $I_{V\;}^{2}$ and *PGA* at individual sites, with seemingly identical slope parameters of the linear models in four different sites (stations ATH, ATHU, LOUT and LTK). This similarity bears hope for establishing an on-site hazard estimator. In addition, for a source located 70 km away from Athens, close to Loutraki (LOUT and LTK stations), we investigated a scaling relation to estimating *PGA* in Athens (stations ATH and ATHU). In this case, correlation is moderate (around 0.50) and further work is required to establish both the observational and physical components that govern a potential inter-site system.

Summarizing, we can state that the high level of seismic hazard and endangered infrastructure in the area mandates the thorough assessment of any developed EEWS and its capabilities. Local industrial activity will benefit greatly from a system that broadcasts alerts, even short-term, to sensitive infrastructure (e.g., oil refineries and power plants). In this aspect, a system that avoids the conundrum of a rapid and accurate epicenter and magnitude estimation could be proven invaluable for automating damage mitigation protocols. Thus, the on-site and inter-site EEWS approaches can issue an alert rapidly and estimate a potential damage level within very few seconds from the origin of the seismic event, increasing the lead time and reducing the blind zone. In any case, further work is required to establish the spatial (and possibly temporal, connected to the evolution of the estimation as seen by different t_w_) behavior of $I_{V\;}^{2}$ and explore its stability both in practical (by estimations in other areas) and theoretical terms. A model documenting its spatial variation is necessary for operational use. Further investigation should focus on integrating observations from events in a wider range of magnitudes, including areas where magnitudes greater than 6.0 are present.

Finally, we note that further work is required in order to define the limitations [65] and uncertainties of a future EEWS as that of statistical independence of the displacement measurements at each station [23], the physical bounds of how accurately the strength of shaking can be estimated [22] along with appropriate metrics to validate the performance of an EEWS [23] contributing to the final goal of near real-time damage estimation [66].

## Figures and Tables

**Table 1:** The average values of ration M_pred_/M_obs_ and the deviation M_pred_-M_obs_ for the t_w_ used.

t_w_	M_pred_/M_obs_	M_pred_-M_obs_
3 s	1.017 ± 0.18	0.05 ± 0.61
4 s	1.012 ± 0.17	0.034 ± 0.59
5 s	1.022 ± 0.19	0.07 ± 0.65

**Figure A1:**
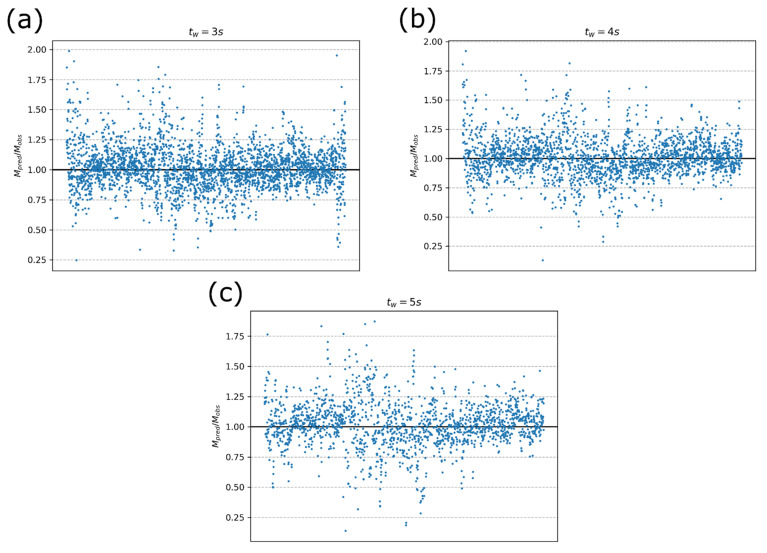
Predicted (M_pred_) to observed (M_obs_) magnitude ratio for the whole dataset, for signal windows of 3 s (**a**), 4 s (**b**) and 5 s (**c**).

**Figure A2:**
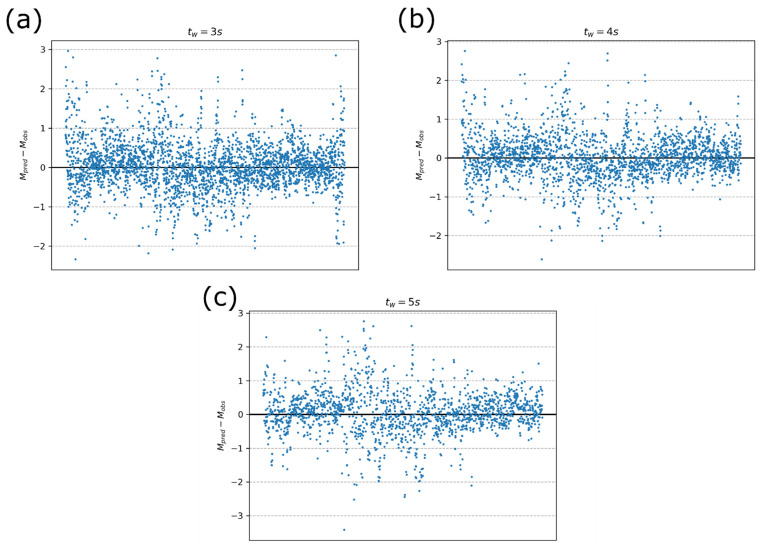
Residuals between predicted (M_pred_) and observed (M_obs_) magnitudes for the whole dataset, for signal windows of 3 s (**a**), 4 s (**b**) and 5 s (**c**).

## Data Availability

Waveform data and station metadata used are publicly available at the EIDA node at GI-NOA (https://eida.gein.noa.gr/, accessed on 15 March 2021). The event catalog used in this study was retrieved from the EIDA node of ISC hosted by the Incorporated Research Institutions for Seismology (IRIS; http://isc-mirror.iris.washington.edu).

## Supplementary Materials

The following are available online at https://www.mdpi.com/article/10.3390/s21155084/s1, The file SUPL_1_Pd includes regression results for the relation: P_d_ = a + blogH + cM, while the file SUPL_2_PGA_REVISED includes regression results for the relation: log(PGA) =a + b log($I_{V\;}^{2}$).

## Appendix A. Relation between Predicted and Observed Magnitudes

Plots of the distribution of the ratio M_pred_/M_obs_ around one (Figure A1) and the deviation M_pred_-M_obs_ around zero (Figure A2 are presented for the used three time-windows starting from the P arrival (i.e., t_w_ 3 s, 4 s, and 5 s).

## References

[1] Kanamori H., Hauksson E., Heaton T. (1997). Real-time seismology and earthquake hazard mitigation. Nature.

[2] Nakamura Y. On the Urgent Earthquake Detection and Alarm System (UrEDAS). Proceedings of the 9th World Conference on Earthquake Engineering.

[3] Gasparini P., Manfredi G., Zschau J. (2011). Earthquake early warning as a tool for improving society’s resilience and crisis response. Soil Dyn. Earthq. Eng..

[4] Parolai S., Bindi D., Boxberger T., Milkereit C., Fleming K., Pittore M. (2015). On-site early warning and rapid damage forecasting using single stations: Outcomes from the REAKT project. Seismol. Res. Lett..

[5] Allen R.M., Gasparini P., Manfredi G., Zschau J. (2007). The Elarms Earthquake Early Warning Methodology and its Application across California. Earthquake Early Warning System.

[6] Allen R.M., Kanamori H. (2003). The potential for earthquake early warning in Southern California. Science.

[7] Kanamori H. (2005). Real-time seismology and earthquake damage mitigation. Annu. Rev. Earth Planet. Sci..

[8] Hloupis G., Vallianatos F. (2015). Wavelet-Based Methods for Rapid Calculations of Magnitude and Epicentral Distance: An Application to Earthquake Early Warning System. Pure Appl. Geophys..

[9] Simons F.J., Dando B., Allen R.M. (2006). Automatic detection and rapid determination of earthquake magnitude by wavelet multiscale analysis of the primary arrival. Earth Planet. Sci. Lett..

[10] Hloupis G., Vallianatos F. (2013). Wavelet-based rapid estimation of earthquake magnitude oriented to early warning. IEEE Geosci. Remote. Sens. Lett..

[11] Espinosa-Aranda J., Jiménez A., Ibarrola G., Alcantar F., Aguilar A., Inostroza M., Maldonado S. (1995). Mexico City seismic alert system. Seism. Res. Lett..

[12] Wu Y.M., Shin T.C., Tsai Y.B. (1998). Quick and reliable determination of magnitude for seismic early warning. Bull. Seism. Soc. Am..

[13] Wu Y.M., Chung J.K., Shin T.C., Hsiao N.C., Tsai Y.B., Lee W.H.K., Teng T.L. (1999). Development of an integrated seismic early warning system in Taiwan- case for Hualien earthquakes. Terr. Atmos. Ocean. Sci..

[14] Wu Y.M., Lee W.H.K., Chen C.C., Shin T.C., Teng T.L., Tsai Y.B. (2000). Performance of the Taiwan Rapid Earthquake Information Release System (RTD) during the 1999 Chi-Chi (Taiwan) earthquake. Seismol. Res. Lett..

[15] Chung A.I., Meier M.A., Andrews J., Böse M., Crowell B.W., McGuire J.J., Smith D.E. (2020). ShakeAlert earthquake early warning system performance during the 2019 Ridgecrest earthquake sequence. Bull. Seismol. Soc. Am..

[16] Iannacone G., Zollo A., Elia L., Convertito V., Satriano C., Martino C., Festa G., Lancieri M., Bobbio A., Stabile T.A. (2010). A prototype system for earthquake early-warning and alert management in southern Italy. Bull. Earthq. Eng..

[17] Brondi P., Picozzi M., Emolo A., Zollo A., Mucciarelli M. (2015). Predicting the macroseismic intensity from early radiated P wave energy for on-site earthquake early warning in Italy. J. Geophys. Res. Solid Earth.

[18] Wu Y.M., Kanamori H. (2005). Experiment of an on-site method for the Taiwan Early Warning System. Bull. Seismol. Soc. Am..

[19] Trugman D.T., Page M.T., Minson S.E., Cochran E.S. (2019). Peak Ground Displacement Saturates Exactly When Expected: Implications for Earthquake Early Warning. J. Geophys. Res. Solid Earth.

[20] Allen R.M., Melgar D. (2019). Earthquake Early Warning: Advances, Scientific Challenges, and Societal Needs. Annu. Rev. Earth Planet. Sci..

[21] Kodera Y., Yamada Y., Hirano K., Tamaribuchi K., Adachi S., Hayashimoto N., Morimoto M., Nakamura M., Hoshiba M. (2018). The Propagation of Local Undamped Motion (PLUM) Method: A Simple and Robust Seismic Wavefield Estimation Approach for Earthquake Early Warning. Bull. Seismol. Soc. Am..

[22] Minson S.E., Baltay A.S., Cochran E.S., Hanks T.C., Page M.T., McBride S.K., Milner K.R., Meier M.-A. (2019). The Limits of Earthquake Early Warning Accuracy and Best Alerting Strategy. Sci. Rep..

[23] Meier M.A. (2017). How “good” are real-time ground motion predictions from Earthquake Early Warning systems?. J. Geophys. Res. Solid Earth.

[24] Burton P.W., Xu Y., Tselentis G.A., Sokos E., Aspinall W. (2003). Strong ground acceleration seismic hazard in Greece and neighboring regions. Soil Dyn. Earthq. Eng..

[25] Giardini D., Wössner J., Danciu L. (2014). Mapping Europe’s seismic hazard. Eos.

[26] Velazquez O., Pescaroli G., Cremen G., Galasso C. (2020). A Review of the Technical and Socio-Organizational Components of Earthquake Early Warning Systems. Front. Earth Sci..

[27] Vavlas N.A., Kiratzi A., Roumelioti Z. (2021). Source Process-Related Delays in Earthquake Early Warning for Example Cases in Greece. Bull. Seismol. Soc. Am..

[28] Sokos E., Tselentis G.-A., Paraskevopoulos P., Serpetsidaki A., Stathopoulos-Vlamis A., Panagis A. (2016). Towards earthquake early warning for the Rion-Antirion bridge, Greece. Bull. Earthq. Eng..

[29] Kapetanidis V., Papadimitriou P., Kaviris G. (2019). Earthquake Early Warning application in Central Greece. Bull. Geol. Soc. Greece.

[30] King G.C.P., Ouyang Z.X., Papadimitriou P., Deschamps A., Gagnepain J., Houseman G., Jackson J.A., Soufleris C., Virieux J. (1985). The evolution of the Gulf of Corinth (Greece): An aftershock study of the 1981 earthquakes. Geophys. J. Int..

[31] Ganas A., Oikonomou I.A., Tsimi C. (2013). NOAfaults: A digital database for active faults in Greece. Bull. Geol. Soc. Greece.

[32] Papadopoulos G.A., Ganas A., Pavlides S. (2002). The problem of seismic potential assessment: Case study of the unexpected earthquake of 7 September 1999 in Athens, Greece. Earth Planets Space.

[33] Papadimitriou P., Voulgaris N., Kassaras I., Kaviris G., Delibasis N., Makropoulos K. (2002). The M_w_ = 6.0, 7 September 1999 Athens earthquake. Nat. Hazards.

[34] Ganas A., Papadopoulos G., Pavlides S.B. (2001). The 7 September 1999 Athens 5.9 Msearthquake: Remote sensing and digital elevation model inputs towards identifying the seismic fault. Int. J. Remote Sens..

[35] Lekkas E. (2001). The Athens earthquake (7 September 1999): Intensity distribution and controlling factors. Eng. Geol..

[36] Kapetanidis V., Karakonstantis A., Papadimitriou P., Pavlou K., Spingos I., Kaviris G., Voulgaris N. (2020). The 19 July 2019 earthquake in Athens, Greece: A delayed major aftershock of the 1999 Mw = 6.0 event, or the activation of a different structure?. J. Geodyn..

[37] Kouskouna V., Ganas A., Kleanthi M., Kassaras I., Sakellariou N., Sakkas G., Valkaniotis S., Manousou E., Bozionelos G., Tsironi V. (2021). Evaluation of macroseismic intensity, strong ground motion pattern and fault model of the 19 July 2019 M_w_ 5.1 earthquake west of Athens. J. Seismol..

[38] Elias P., Spingos I., Kaviris G., Karavias A., Gatsios T., Sakkas V., Parcharidis I. (2021). Combined Geodetic and Seismological Study of the December 2020 M_w_ = 4.6 Thiva (Central Greece) Shallow Earthquake. Appl. Sci..

[39] Kaviris G., Spingos I., Millas C., Kapetanidis V., Fountoulakis I., Papadimitriou P., Voulgaris N., Drakatos G. (2018). Effects of the January 2018 seismic sequence on shear-wave splitting in the upper crust of Marathon (NE Attica, Greece). Phys. Earth Planet. Inter..

[40] Makropoulos K., Kaviris G., Kouskouna V. (2012). An updated and extended earthquake catalogue for Greece and adjacent areas since 1900. Nat. Hazards Earth Syst. Sci..

[41] Papazachos B.C., Papazachou C. (2003). The Earthquakes of Greece.

[42] Papazachos B.C., Comninakis P.E., Papadimitriou E.E., Scordilis E.M. (1984). Properties of the February-March 1981 seismic sequence in the Alkyonides gulf of central Greece. Ann. Geophys..

[43] Collier R.E.L., Pantosti D., D’Addezio G., De Martini P.M., Masana E., Sakellariou D. (1998). Paleoseismicity of the 1981 Corinth earthquake fault: Seismic contribution to extensional strain in central Greece and implications for seismic hazard. J. Geophys. Res. Solid Earth.

[44] Scordilis E.M. (2006). Empirical Global Relations Converting Ms and mb to Moment Magnitude. J. Seismol..

[45] Kaviris G., Papadimitriou P., Makropoulos K. (2007). Magnitude Scales in Central Greece. Bull. Geol. Soc. Greece.

[46] Evangelidis C., Triantafyllis N., Samios M., Boukouras K., Kontakos K., Ktenidou O.J., Fountoulakis I., Kalogeras I., Melis N., Galanis O. (2021). Seismic Waveform Data from Greece and Cyprus: Integration, Archival, and Open Access. Seismol. Res. Lett..

[47] Krischer L., Megies T., Barsch R., Beyreuther M., Lecocq T., Caudron C., Wassermann J. (2015). ObsPy: A bridge for seismology into the scientific Python ecosystem. Comput. Sci. Discov..

[48] Baer M., Kradolfer U. (1987). An automatic phase picker for local and teleseismic events. Bull. Seismol. Soc. Am..

[49] Crotwell H.P., Owens T.J., Ritsema J. (1999). The TauP Toolkit: Flexible Seismic Travel-time and Ray-path Utilities. Seismol. Res. Lett..

[50] Rigo A., Lyon-Caen H., Armijo R., Deschamps A., Hatzfeld D., Makropoulos K., Papadimitriou P., Kassaras I. (1996). A microseismic study in the western part of the Gulf of Corinth (Greece): Implications for large-scale normal faulting mechanisms. Geophys. J. Int..

[51] Wu Y.-M., Kanamori H. (2008). Development of an Earthquake Early Warning System Using Real-Time Strong Motion Signals. Sensors.

[52] Wu Y.M., Zhao L. (2006). Magnitude estimation using the first three seconds *P*-wave amplitude in earthquake early warning. Geophys. Res. Lett..

[53] Zollo A., Lancieri M., Nielsen S. (2006). Earthquake magnitude estimation from peak amplitudes of very early seismic signals on strong motion, Geophys. Res. Lett..

[54] Zollo A., Amoroso O., Lancieri M., Wu Y.M., Kanamori H. (2010). A threshold-based earthquake early warning using dense accelerometer networks. Geophys. J. Int..

[55] Nof R.N., Allen R. (2016). Implementation the ElarmS Earthquake Early Warning Algorithm on the Israeli Seismic Network. Bull. Seismol. Soc. Am..

[56] Sadeh M., Ziv A., Wust-Bloch H. (2014). Real-time magnitude proxies for earthquake early warning in Israel. Geophys. J. Int..

[57] Wu Y.M., Kanamori H., Allen R., Hauksson E. (2007). Determination of earthquake early warning parameters, *τ_c_* and *P_d_*, for southern California. Geophys. J. Int..

[58] Olson E.L., Allen R.M. (2005). The deterministic nature of earthquake rupture. Nature.

[59] Lockman A., Allen R.M. (2007). Magnitude-period scaling relations for Japan and the Pacific Northwest: Implications for earthquake early warning. Bull. Seismol. Soc. Am..

[60] Allen R.M., Gasparini P., Kamigaichi O., Böse M. (2009). The status of earthquake early warning around the world: An introductory overview. Seismol. Res. Lett..

[61] Lior I., Ziv A., Madariaga R. (2016). P-Wave Attenuation with Implications for Earthquake Early Warning. Bull. Seismol. Soc. Am..

[62] Festa G., Zollo A., Lancieri M. (2008). Earthquake magnitude estimation from early radiated energy. Geophys. Res. Lett..

[63] Satriano C., Wu Y.H., Zollo A., Kanamori H. (2011). Earthquake early warning: Concepts, methods and physical grounds. Soil Dyn. Earthq. Eng..

[64] Colombelli S., Caruso A., Zollo A., Festa G., Kanamori H. (2015). A *P* wave-based, on-site method for earthquake early warning. Geophys. Res. Lett..

[65] Wald D.J. (2020). Practical limitations of earthquake early warning. Earthq. Spectra.

[66] Scaini C., Petrovic B., Tamro A., Moratto L., Parolai S. (2021). Near-Real-Time damage estimation for Building based on strong motion recordings: An application to target areas in Northeastern Italy. Seismol. Res. Lett..

[67] Van Der Walt S., Colbert S.C., Varoquaux G. (2011). The NumPy array: A structure for efficient numerical computation. Comput. Sci. Eng..

[68] Hunter J.D. (2007). Matplotlib: A 2D Graphics Environment. Comput. Sci. Eng..

